# STAT3 plays an important role in DNA replication by turning on WDHD1

**DOI:** 10.1186/s13578-020-00524-x

**Published:** 2021-01-07

**Authors:** Yunying Zhou, Jason J. Chen

**Affiliations:** 1 Medical Research & Laboratory Diagnostic Center, Jinan Central Hospital, Cheeloo College of Medicine, Shandong University, Jinan, Shandong China; 2 Department of Microbiology, School of Basic Medical Sciences, Cheeloo College of Medicine, Shandong University, Jinan, Shandong China; 3 Medical Research & Laboratory Diagnostic Center, Central Hospital Affiliated To Shandong First Medical University, Jinan, China; 4 The Cancer Research Center, Cheeloo College of Medicine, Shandong University, Jinan, Shandong China

**Keywords:** STAT3, WDHD1, Transcription, DNA replication

## Abstract

**Background:**

Signal transducers and activators of transcription 3 (STAT3) is a transcription factor that plays a key role in many cellular processes such as cell growth and cancer. However, the functions and mechanisms by which STAT3 regulates cellular processes are not fully understood.

**Results:**

Here we describe a novel function of STAT3. We demonstrated that STAT3 plays an important role in DNA replication. Specifically, knockdown of STAT3 reduced DNA replication while activation and ectopic expression of STAT3 promoted DNA replication. We further identified the WD repeat and HMG-box DNA-binding protein 1 (WDHD1), which plays an important role in DNA replication initiation, as a novel STAT3 target gene that mediated the DNA replication function of STAT3. We showed that STAT3 bind the promoter/up regulatory region of WDHD1 gene.

**Conclusions:**

These studies identified a novel function of STAT3 that is mediated by its newly identified target gene WDHD1 and have important implications.

## Background

Signal transducers and activators of transcription (STAT) family members are transcription factors that mediate many cellular processes and involved in the pathogenesis of various human diseases [1]. STAT3 is a core member of the STAT protein family and plays a key role in many critical cellular processes such as proliferation, differentiation, survival, immunosuppression, angiogenesis and tumorigenesis [2, 3]. Targeted disruption of the mouse Stat3 gene leads to early embryonic lethality [4]. STAT3 is essential for the differentiation of the TH17 helper T cells [5]. STAT3 is aberrantly activated in a variety of tumors, STAT3 signaling promotes cancer through inflammation, obesity, stem cells and the pre-metastatic niche [3, 6].

STAT3 activation is triggered primarily by interleukin 6 (IL-6) family cytokine receptor-associated Janus kinases (JAKs) [7, 8] as well as receptor tyrosine kinases such as epidermal growth factor receptor (EGFR) [9–12], and non-receptor tyrosine kinases such as SRC [13–16]; Phosphorylation of STAT3 on residue Y705 induces dimerization and results in its nuclear translocation and activation of the transcriptional regulator function. STAT3 may be further modified by phosphorylation on a serine residue (S727) to promote its full activation [17, 18]. Kinases responsible for STAT3 phosphorylation at S727 include the MAPK cascade [18]. IL-6 also increases the expression of STAT3 gene [19]. Toll-like receptors (TLRs) and microRNAs were recently identified to regulate JAK-STAT signaling in cancer [3, 20, 21].

Activated STAT3 can up-regulate the transcription of numerous genes, many of these genes are its direct target [1, 22]. STAT3 target genes include cyclin D1 [23], BclXL [24], c-Myc [25, 26], β-catenin [27], nuclear factor-κB (NF-κB) [28]. In addition to its established role as a transcription factor in cancer, STAT3 regulates mitochondrion functions [29, 30] as well as gene expression through epigenetic mechanisms [3]. The process of DNA replication initiation consists of two steps: pre-replicative complex (pre-RC) assembly and activation [31]. WDHD1 (WD repeat and HMG-box DNA-binding protein 1) plays a role in both pre-RC assembly [32] and pre-RC activation [32–34]. WDHD1 is localized adjacent to replication foci, interacts with human primase-DNA polymerase/Mcm10 and is required for DNA synthesis [33, 35, 36]. A role for WDHD1 in G1 checkpoint control has recently been suggested [36, 37]. We have recently shown that WDHD1 plays a role in viral oncogene-induced re-replication [37].

In this report we described a novel biological function of STAT3. We showed that STAT3 played an important role in DNA replication. In addition, we identified WDHD1 as a STAT3 regulated target gene that mediated the DNA replication function of STAT3.

## Results

### STAT3 plays a role in DNA replication

**Fig. 1:**
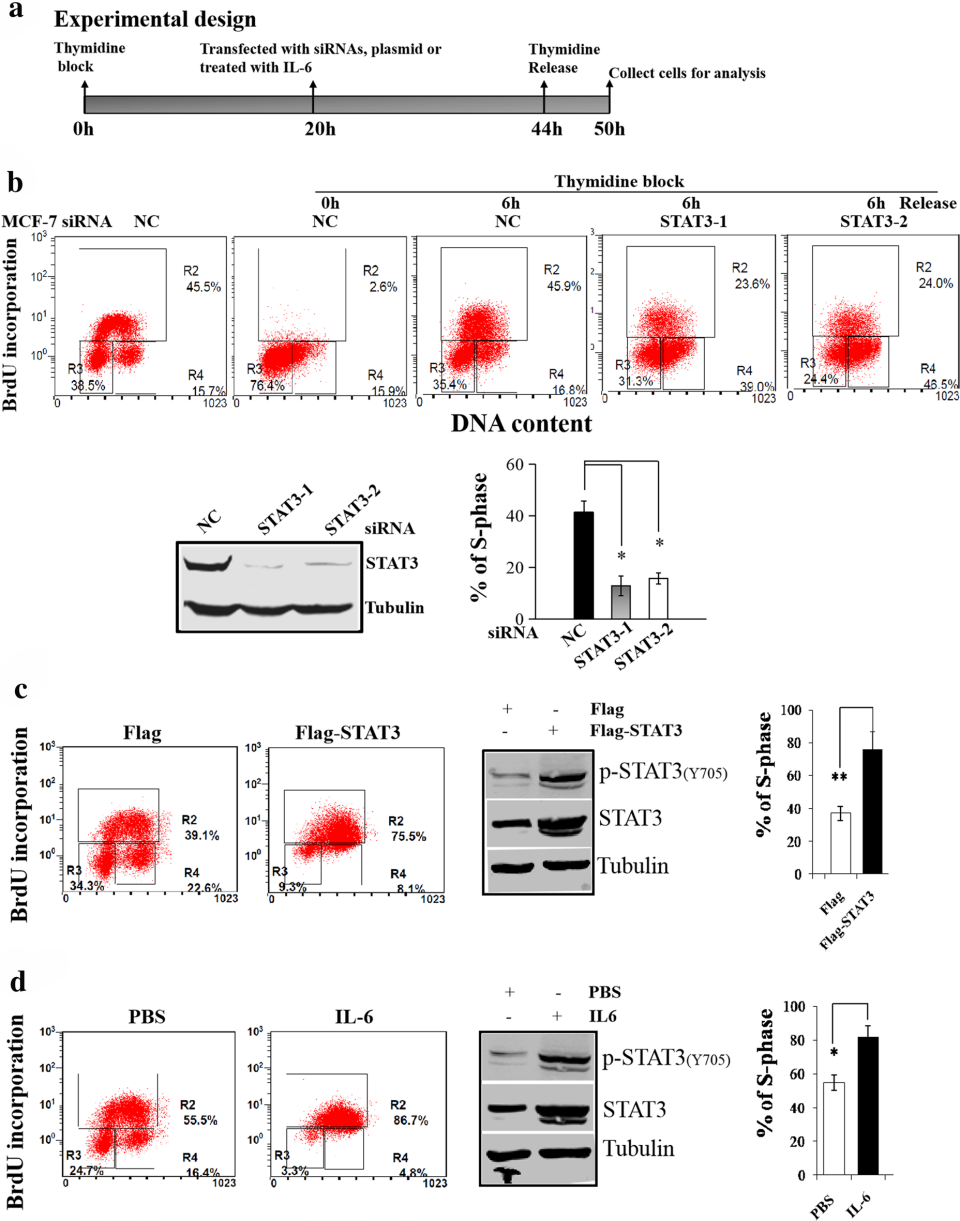
STAT3 plays a role in DNA replication. **a** Experimental design. **b-d** After thymidine block, MCF-7 cells were transfected with siRNAs or plasmids, or treated with IL-6. After releasing, cells were stained with BrdU and analyzed by flow cytometry. Western blots were performed using transfected cell extracts without thymidine treatment. A representative experiment of 3 was shown. **b** siRNA transfection. **c** Plasmids transdaction. **d** IL-6 treatment

Our recent studies demonstrated that both STAT3 and WDHD1 were up-regulated in oncogene overexpressing cells [37]. STAT family proteins recognize a consensus DNA binding motif of TTCC (C or G) GGAA (or generically TTN5AA) [38]. There are three putative STAT3 binding sites in the WDHD1 promoter/up regulatory region. Since WDHD1 plays a role in DNA replication, we reasoned that STAT3 might be involved in DNA replication by up-regulating WDHD1. To test this possibility, we knocked down STAT3 in MCF-7 cells by siRNAs and measured BrdU incorporation (Fig. 1). Since STAT3 knock-down could lead to cell arrest at the G1 phase of the cell cycle [39–41] and thus reduce BrdU incorporation, we synchronized cells at late G1/early S-phase by thymidine block before siRNA transfection and the effects of thymidine block and release of cell cycle were demonstrated in Additional file 1: Fig. 1a and b. DNA replication was measured after releasing from thymidine block (Fig. 1a and b). Notably, knock-down of STAT3 by siRNAs significantly reduced BrdU incorporation in MCF-7 cells, indicating that STAT3 plays a role in DNA replication (Fig. 1b). Similar results were observed in HeLa cells (Additional file 2: Fig. 2).

Next we examined the extent to which ectopic expression of STAT3 would facilitate DNA replication. For this, we transfected MCF-7 cells with plasmid encoding STAT3. To avoid the potential effect of STAT3 on G1/S phase transition, cells were thymidine blocked before transfection. After transfection and releasing from the block, DNA replication was measured. As shown in Fig. 1c, STAT3 expression was significantly increased after transfection with the STAT3 plasmid (left panel), and DNA replication was also significantly increased (Fig. 1c and quantified in the lower right panel). We also examined whether treatment of cells by the physiological activator of STAT3 could increase DNA replication. For this, thymidine blocked MCF-7 cells were treated with IL-6 and BrdU incorporation was measured (Fig. 1d). As expected, IL-6 treatment led to an increase in both steady-state level and phosphorelated form of STAT3 (Upper left panel), more importantly, DNA replication was also significantly increased (Fig. 1c and quantified in the lower right panel).

### WDHD1 expression is regulated by STAT3

**Fig. 2:**
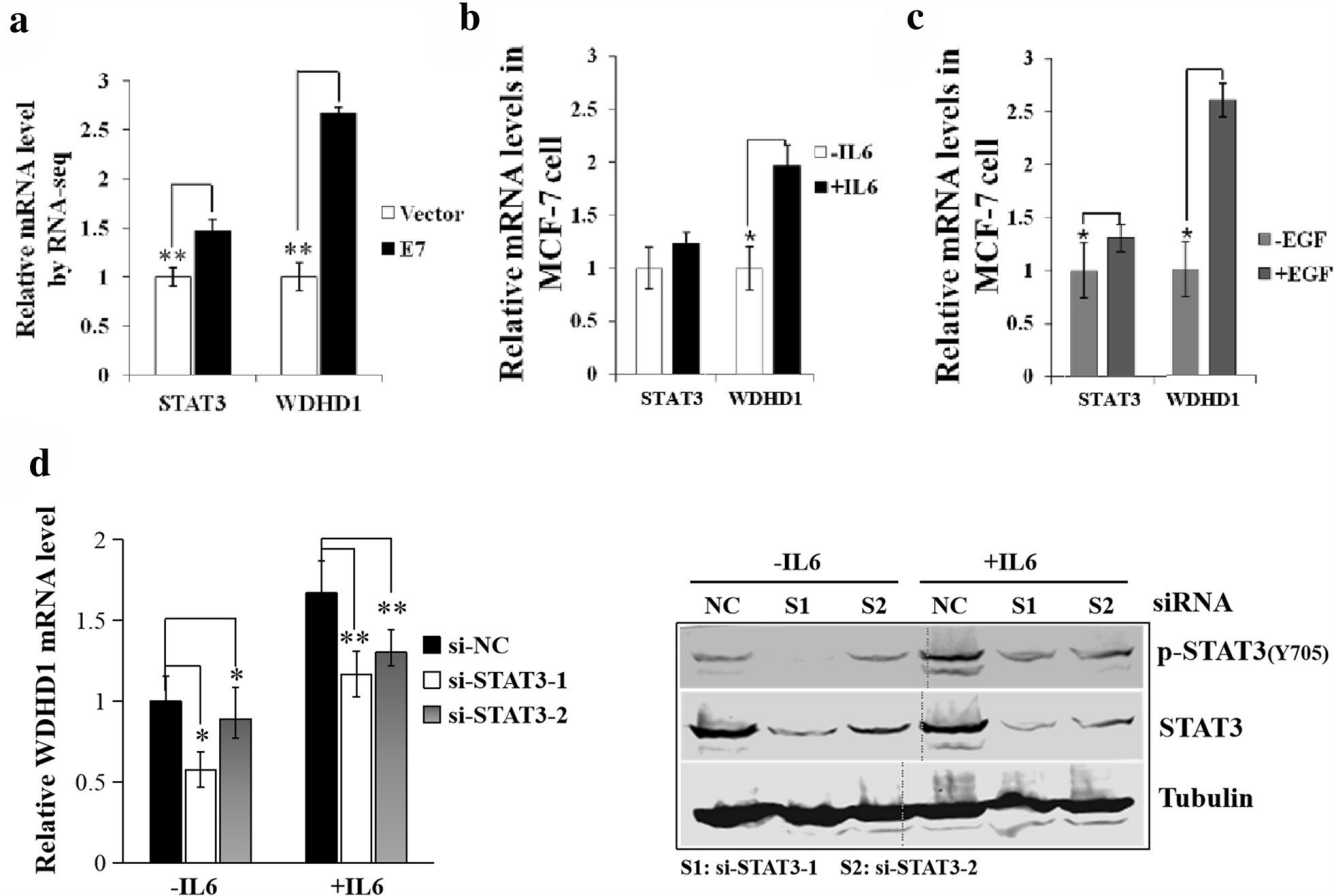
WDHD1 expression is regulated by STAT3. **a** STAT3 and WDHD1 mRNA levels by RNA-seq. **b** STAT3 and WDHD1 mRNA levels in IL-6 treated MCF-7 cells determined by real-time-PCR analysis. **c** STAT3 and WDHD1 mRNA levels in EGF treated MCF-7 cells determined by real-time-PCR analysis. **d** WDHD1 mRNA levels in IL-6 treated, STAT3 siRNA transfected MCF-7 cells were determined by real-time-PCR analysis (Left panel). The steady-state levels of STAT3 were determined by Western blot (Right panel). Data from a representative experiment of 3 were shown. Error bars reflect the standard deviations of the mean. *p < 0.05, ** p < 0.01. *NC* negative control

Having demonstrated a role for STAT3 in DNA replication, we wanted to explore the mechanism by which STAT3 facilitates replication. Since STAT3 is a transcription factor, it is likely that STAT3 facilitates DNA replication by regulating a gene whose product involves in DNA replication. WDHD1 is certainly a potential candidate to mediate DNA replication function for STAT3. We therefore investigated the possibility that WDHD1 is a transcriptional target gene for STAT3. As mentioned and shown, both STAT3 and WDHD1 were up-regulated in HPV oncogene E7 expressing cells (Fig. 2a). Upon treating MCF-7 cells with STAT3 activator IL-6, WDHD1 mRNA level went up (by 2.3-fold) (Fig. 2b). When treated with EGF, another growth factor known to activate STAT3, WDHD1 mRNA level also went up (by 1.4-fold) (Fig. 2c). Similar results were obtained in HeLa cells (Additional file 3: Fig. 3). Significantly, the WDHD1 mRNA levels in IL-6-treated cells were significantly down-regulated upon transfection with STAT3 siRNAs (Fig. 2d). Thus, we conclude that WDHD1 mRNA level is regulated by STAT3.

**Fig. 3:**
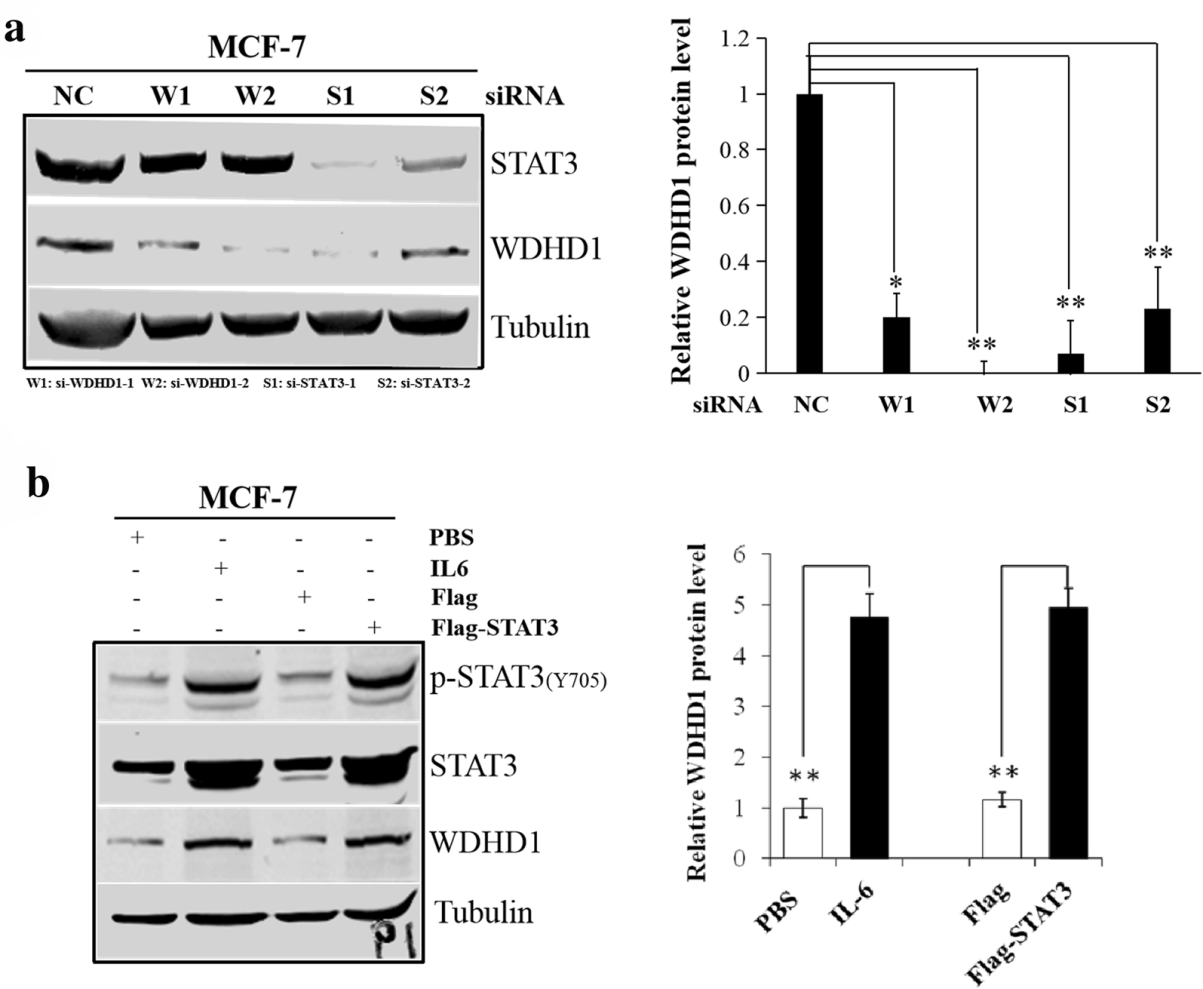
The steady-state level of WDHD1 is regulated by STAT3. **a** The WDHD1 protein levels in MCF-7 cells were examined by Western blotting after siRNA transfection (Left panel). Data were summarized. **b** The WDHD1 protein levels in MCF-7 cells were examined by Western blotting after IL-6 treatment or transfection with STAT3 plasmid (Left panel). Data were summarized (Right panel). Data from a representative experiment of 3 were shown. Error bars reflect the standard deviations of the mean. *p < 0.05, **p < 0.01. *NC* negative control

Next we examined whether the steady-state levels of WDHD1 protein is subjected to STAT3 regulation. As shown in Fig. 3a and consistent with mRNA levels, knockdown of STAT3 by siRNAs reduced the steady-state levels of WDHD1 protein (14-fold by si-STAT3-1 and 4-fold for si-STAT3-2) in MCF-7 cells (Fig. 3a). In contrast, siRNA knockdown of WDHD1 did not lead to a reduction in the steady-state levels of STAT3. Similar results were observed in HeLa cells (Additional file 4: Fig. 4). On the other hand, overexpression of STAT3 increased the steady-state levels of WDHD1 protein (Fig. 3b). Furthermore, IL-6 treatment also led to an up-regulation of WDHD1 protein levels (Fig. 3b). These results further support the notion that WDHD1 is a target for STAT3.

### STAT3 binds to the WDHD1 promoter/up regulatory region

**Fig. 4:**
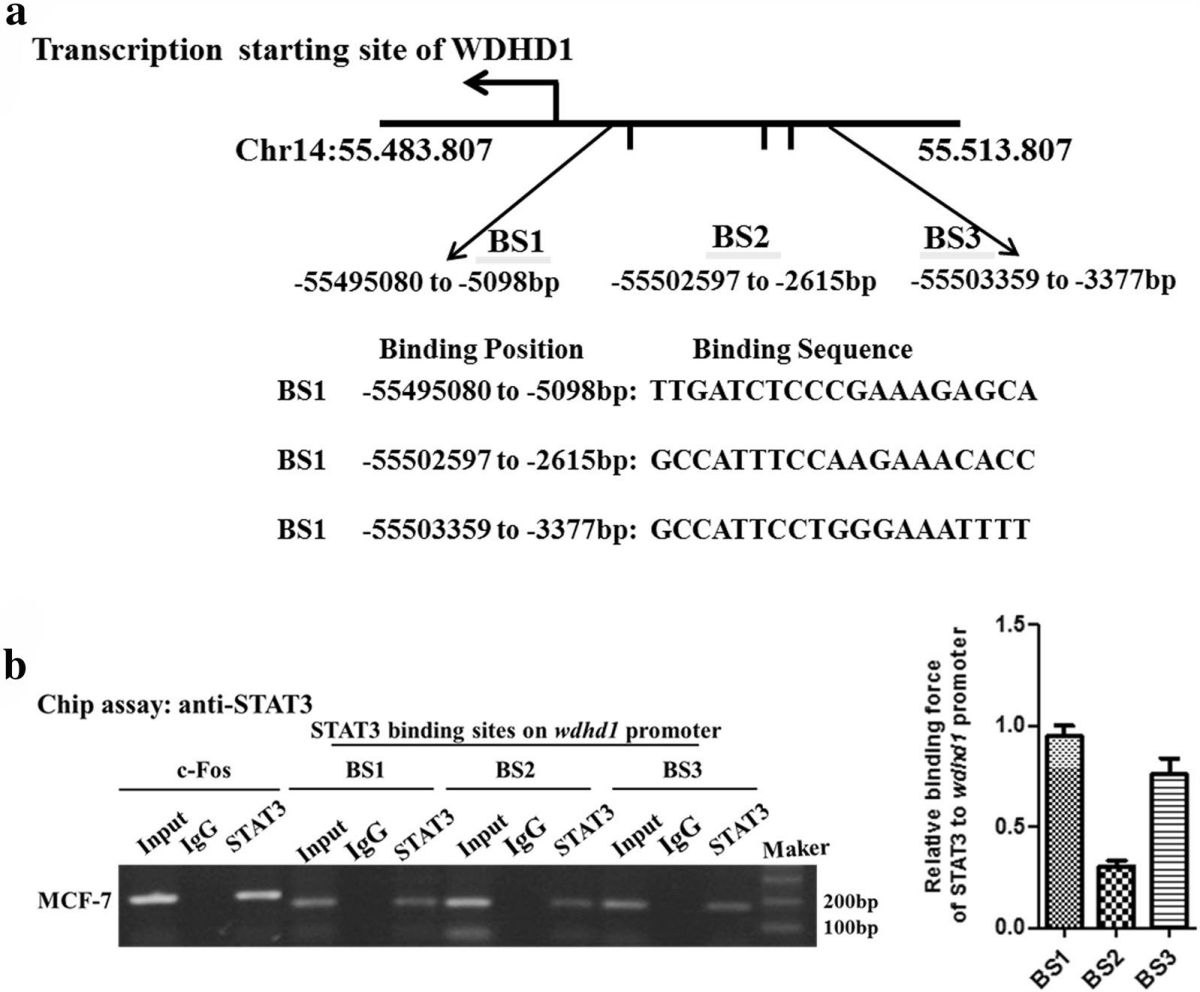
STAT3 binds to *WDHD1* promoter/up regulatory region region. **a** Three putative STAT3 binding sites (SB), named SB1 to SB3, were identified in the WDHD1 promoter/up regulatory region. **b** Immunoprecipitations were performed using anti-STAT3 or control IgG antibodies. PCR was performed with c-Fos or WDHD1 primers. Data from a representative experiment of 3 were shown (Left panel) and summarized (Right panel)

To further establish the role of STAT3 in regulating WDHD1 expression, we examined the association of STAT3 at the promoter/up regulatory region of WDHD1. STAT family proteins recognize a consensus DNA binding motif of TTCCC/GGGAA [38]. Three putative STAT3 binding sites, named SB1 to SB3, were identified in the WDHD1 promoter/up regulatory region (Fig. 4a). ChIP assays were performed to examine the potential association of STAT3 with these putative binding sites. c-Fos served as a positive control as STAT3 has been shown by ChIP assay to bind and up-regulate c-Fos expression [42, 43]. Significantly, STAT3 bound to all three of putative binding sites in the wdhd1 promoter/up regulatory region in MCF-7 cells at varying levels (Fig. 4b). It bound SB1 the most efficiently, to SB2 weakly, and to SB3 with reduced efficiency. Interestingly, in HeLa cells, STAT3 bound to SB1, SB3 but not SB2 (Additional file 5: Fig. 5). These results demonstrate an association of STAT3 to the wdhd1 promoter/up regulator region.

### WDHD1 can functionally rescue defect in DNA replication and re-replication caused by STAT3 knockdown

**Fig. 5:**
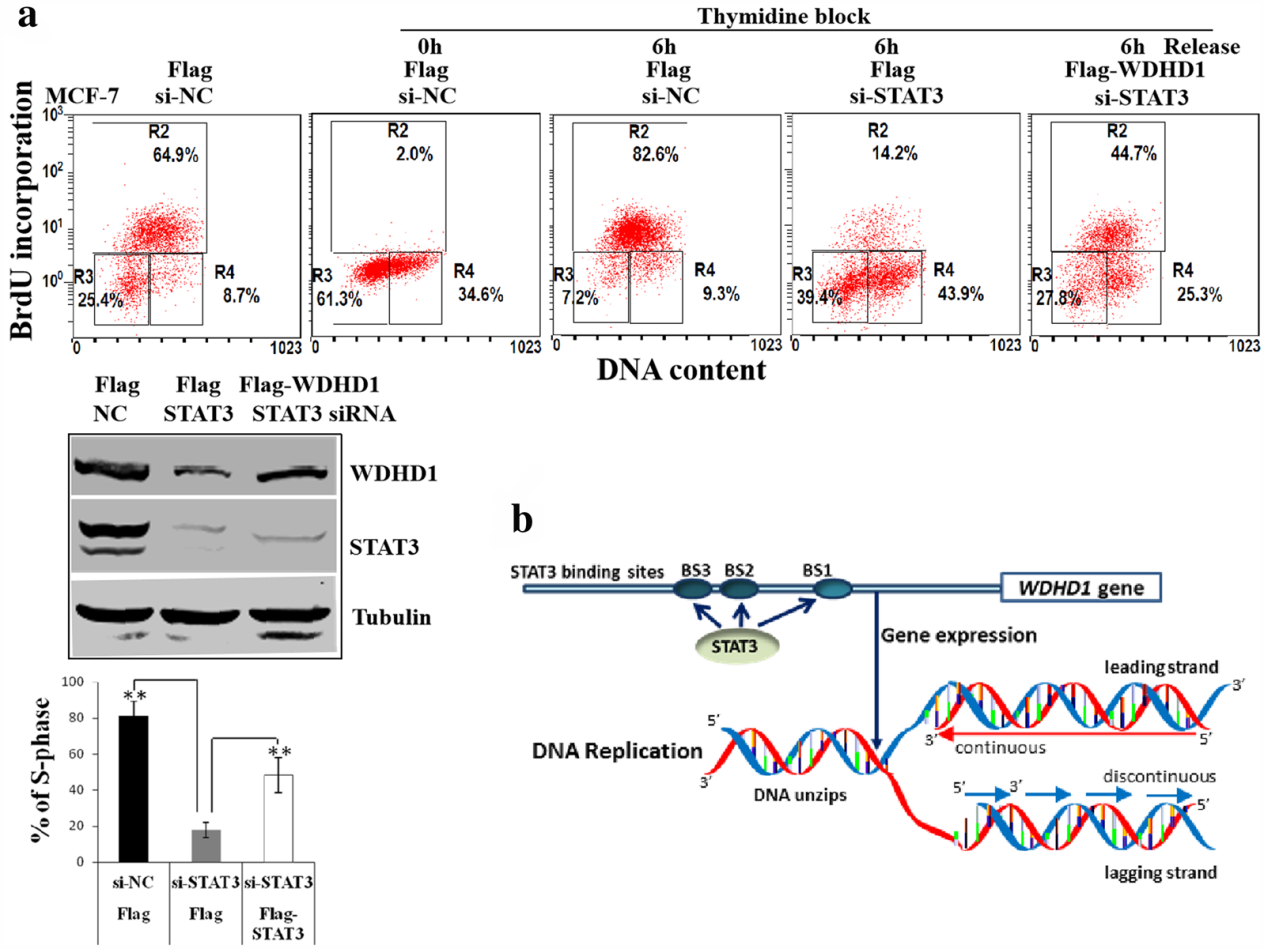
Overexpression of WDHD1 rescued DNA replication reduction induced by STAT3 knocking down. **a** After thymidine block and siRNA transfection, MCF-7 cells were released, transfected with WDHD1 plasmid, stained with BrdU and analyzed by flow cytometry. **a** representative experiment of 3 was shown (Upper panel). The stability of STAT3 and WDHD1 was monitored by immunoblotting analyses (Middle panel). Data were summarized (Lower panel). **b** Proposed model. STAT3 binds to the promoter/regulatory region of WDHD1, turns on its transcription. WDHD1 in turn participates DNA replication

As a STAT3 target functioning in DNA replication, WDHD1 should be able to rescue the DNA replication defect caused by STAT3 knockdown. We therefore overexpressed WDHD1 in cells STAT3 has been knocked down. For this, MCF-7 cells were synchronized at the late G1/early S-phase of the cell cycle with thymidine, transfected with STAT3 targeting siRNAs, then transfected with WDHD1 plasmid, DNA replication was examined. Significantly, expression of WDHD1 rescued DNA replication reduction caused by STAT3 knockdown (from 14.2% to 44.7%, Fig. 5a). These results demonstrate that WDHD1 can functionally replace STAT3 for its DNA replication activity and provide further evidence that WDHD1 is a target for STAT3.

Our data presented in this manuscript suggest a model where STAT3 binds to the promoter/up regulatory region of WDHD1, turns on its transcription. WDHD1 in turn participates DNA replication (Fig. 5b). These studies identified a novel function of STAT3 that is mediated by its target gene WDHD1 and have important implications.

## Discussion

STAT3 mediates many cellular processes and involves in the pathogenesis of various human diseases, including cancer. As a transcription factor, numerous transcriptional targets have been identified for STAT3 [44], these include c-Fos [43], HIF-1α [45] and cyclin D1 [46, 47]. Our data support the notion that STAT3 regulates WDHD1 transcription and therefor promotes DNA replication. Interestingly, both STAT3 and WDHD1 have been implicated in cell cycle checkpoint control, epithelial-mesenchymal transition, tumor growth and metastasis [36, 37, 48, 49] and reviewed in [50, 51]. It would be interesting to examine whether STAT3 performs these functions through WDHD1. In addition to the above described activities, WDHD1 has also been implicated in the post-transcriptional step of the centromeric silencing pathway [52], chromosome congression by regulating the assembly of centromere Protein A (CENP-A) at centromeres [53], homologous recombination repair by regulating DNA end resection [54, 55], and the stability of Histone Acetyltransferase Gcn5 and histone H3 acetylation [56]. As a regulator of WDHD1, it is important to examine whether STAT3 is also involved in these biological processes.

How STAT3 regulates WDHD1 transcription remains to be examined. We have found that STAT3 bound WDHD1 promoter/up regulatory region at three sites. We therefore speculate that STAT3 regulates WDHD1 transcription through direct binding to its promoter/up regulatory region. In addition to act as a traditional transcription factor, STAT3 may regulate WDHD1 expression through epigenetic mechanisms. Future studies will explore this possibility.

A recent study showed that Cdc6 could be induced by STAT3 signaling [57]. As Cdc6 is also play a role in DNA replication initiation, it may contribute to the DNA replication activities identified for STAT3 in this study. Notably, the replication protein A 32 kDa subunit (RPA p32) binds STAT3 and regulates STAT3 transcriptional activity [58]. RPA p32 has important functions in DNA replication. It remains to be explored the extent to which STAT3 performs its DNA replication function through Cdc6 and RPA p32.

## Conclusions

In this study, we provide evidence that STAT3 plays an important role in DNA replication. To the best of our knowledge, this is a first report for a role in DNA replication regarding STAT3. DNA replication is a fundamental activity for a living cell and identification of DNA replication function for STAT3 has important implications.

## Materials and methods

### Cell culture

Human breast cancer MCF-7 cells (American Type Culture Collection (ATCC), HTB-22) and cervical cancer HeLa cells (ATCC, CCL-2) were grown in Dulbecco’s modified Eagle’s medium supplemented with 10% fetal bovine serum (FBS) and incubated at 37 °C in a humidified atmosphere of 5% CO_2_.

### Real-time PCR

Total RNA was isolated with an RNeasy kit (Qiagen) from MCF-7 and HeLa cells and their correspondent targeting siRNA expressing cells according to the manufacturer’s instruction. cDNA was synthesized with a Superscript VILO cDNA synthesis kit (Invitrogen). The iTaq Universal SYBR Green Supermix (Bio-Rad) was used for real-time PCR (qRT-PCR) in the Bio-Rad CFX96 Touch Real-Time PCR Detection system. Data were analyzed using the 2 − ∆∆Ct method.

### Chromatin immunoprecipitation assay

Chromatin immunoprecipitation (ChIP) assay was performed using the ChIP assay kit from Millipore following the manufacturer’s protocol. Immunoprecipitations were performed using anti-STAT3 or control IgG antibodies. PCR was performed with the Simple ChIP Human c-Fos Promoter Primers (Cell Signaling, #4663) that has been shown to interact directly with STAT3 [42, 43] and with the primers designed from the sequences of the human WDHD1 promoter/up regulatory region gene.

### siRNAs and transfection

Cells were transfected with a final concentration of 20 nM siRNA per target gene (Table 1) using Lipofectamine 2000 transfection reagent (Invitrogen) according to the manufacturer’s instructions. For gene knockdown analysis, cells were harvested 48 h post-transfection and specific protein levels were analyzed by immunoblot. For cell cycle analysis, MCF-7 cells were transfected with 20 nM siRNA for 24 h after blocked with thymidine for 20 h.

**Table 1:** The sequence of siRNA duplexes

Gene	Sequence (from 5′-3′)
si-STAT3-1	CAGGGUGUCAGAUCACAUGGGCUAA
si-STAT3-2	GGACGACUUUGAUUUCAACTT
si-WDHD1-1	GCAUGUACCCUAAGAAUAA
si-WDHD1-2	GCAAAGUUAUGGAAAGUAU

### Western-blot

To obtain total protein, cell extraction was prepared in lysis buffer (10 mM Tris [pH 7.4], 1% SDS, and 1.0 mM sodium orthovanadate). The protein concentration was measured by the bicinchoninic acid (BCA) protein assay reagent (Pierce) and confirmed by Coomassie blue staining of membranes after blotting. Equal amounts of protein from each cell lysate were separated in an SDS polyacrylamide gel (PAGE) and transferred onto a nitrocellulose filter membrane (NC) membrane. Membranes were blotted with antibodies against WDHD1 (abcam, ab72436), STAT3 (Cell Signaling, 4904S), Phospho-STAT3 (Tyr705) (Cell Signaling, #9145), and tubulin (Sigma, T-4026).

### Flow cytometry

For the bromodeoxyuridine (BrdU) labeling experiment, BrdU (Final 20 μM) was added to the medium 2 h before collection of cells. Cells were then harvested and fixed in 70% ethanol. The cells were permeabilized with 2 N HCl–0.5% Triton X-100, neutralized with 0.1 M sodium tetraborate, stained with monoclonal anti-BrdU (BD Biosciences), and then with anti-mouse IgG F(ab)2-FITC (Sigma), and counterstained with PBS-7-AAD-RNase A. Flow cytometric analysis was performed on a BD FACSAria™ III sorter instrument equipped with BD FACSDiva™ 7.0 software (BD Biosciences, New Jersey, USA). FITC 490 nm fluorescence was acquired in logarithmic amplification in FL1 and 7-AAD 650 nm fluorescence was acquired in linear amplification in FL3. Cell cycle analysis was done using Cytomics™ FC500 Flow Cytometry CXP 2.0.

### Statistical analysis

Data were presented as means and standard deviations (SDs). The Student’s *t* test was used to compare the differences between means. P < 0.05 was considered significant.

## Supplementary information

**Additional file 1: Figure 1.** Time selection of thymidine block and release of cell cycle. **a** MCF-7 cells were blocked with thymidine for 0h, 20h and 24h. **b** After thymidine block for 20h, MCF-7 cells were released for 1h, 2h, 3h, 4h, 5h, 6h, 8h, 10h.

**Additional file 2: Figure 2.** STAT3 plays a role in DNA replication in HeLa cells. After thymidine block, HeLa cells were transfected with siRNAs targeting STAT3. After releasing, cells were stained with BrdU and analyzed by flow cytometry. Data from a representative experiment of 3 were shown (Upper panel) and summarized (Lower panel). Western blots were performed using transfected cell extracts without thymidine treatment.

**Additional file 3: Figure 3.** WDHD1 mRNA expression is regulated by STAT3 in HeLa cells. STAT3 and WDHD1 mRNA levels in IL-6 or EGF treated HeLa cells determined by real-time-PCR analysis. Data from a representative experiment of 3 were shown. Error bars reflect the standard deviations of the mean. *p < 0.05 **p < 0.01. *NC* negative control.

**Additional file 4: Figure 4.** The steady-state levels of WDHD1 is regulated by STAT3 in HeLa cells. **a** STAT3 and WDHD1 protein levels in HeLa cells were examined by Western blotting after siRNA transfection (Left panel). Data were summarized (Right panel). Data from a representative experiment of 3 were shown. Error bars reflect the standard deviations of the mean. *p < 0.05 **p < 0.01. *NC* negative control.

**Additional file 5: Figure 5.** STAT3 binds to *WDHD1* promoter/up regulatory region in HeLa cells. Immunoprecipitations were performed using anti-STAT3 or control IgG antibodies. PCR was performed with c-Fos or WDHD1 primers. Data from a representative experiment of 3 were shown (Left panel) and summarized (Right panel).

## Data Availability

All datasets generated for this study are included in the article/supplementary material.

## Abbreviations

WDHD1: WD repeat and HMG-box DNA-binding protein 1
STAT: Signal transducers and activators of transcription
IL-6: Interleukin 6
JAKs: Janus kinases
EGFR: Epidermal growth factor receptor
TLRs: Toll-like receptors
NF-κB: Nuclear factor-κB
BrdU: Bromodeoxyuridine
FACS: Fluorescence-activated cell sorting
ChIP: Chromatin immunoprecipitation assay
