# Supplementary figures and images for: STAT3 plays an important role in DNA replication by turning on WDHD1
Source: Cell Biosci. 2021 Jan 7;11:10. doi: 10.1186/s13578-020-00524-x (PMC7792067; doi:10.1186/s13578-020-00524-x)

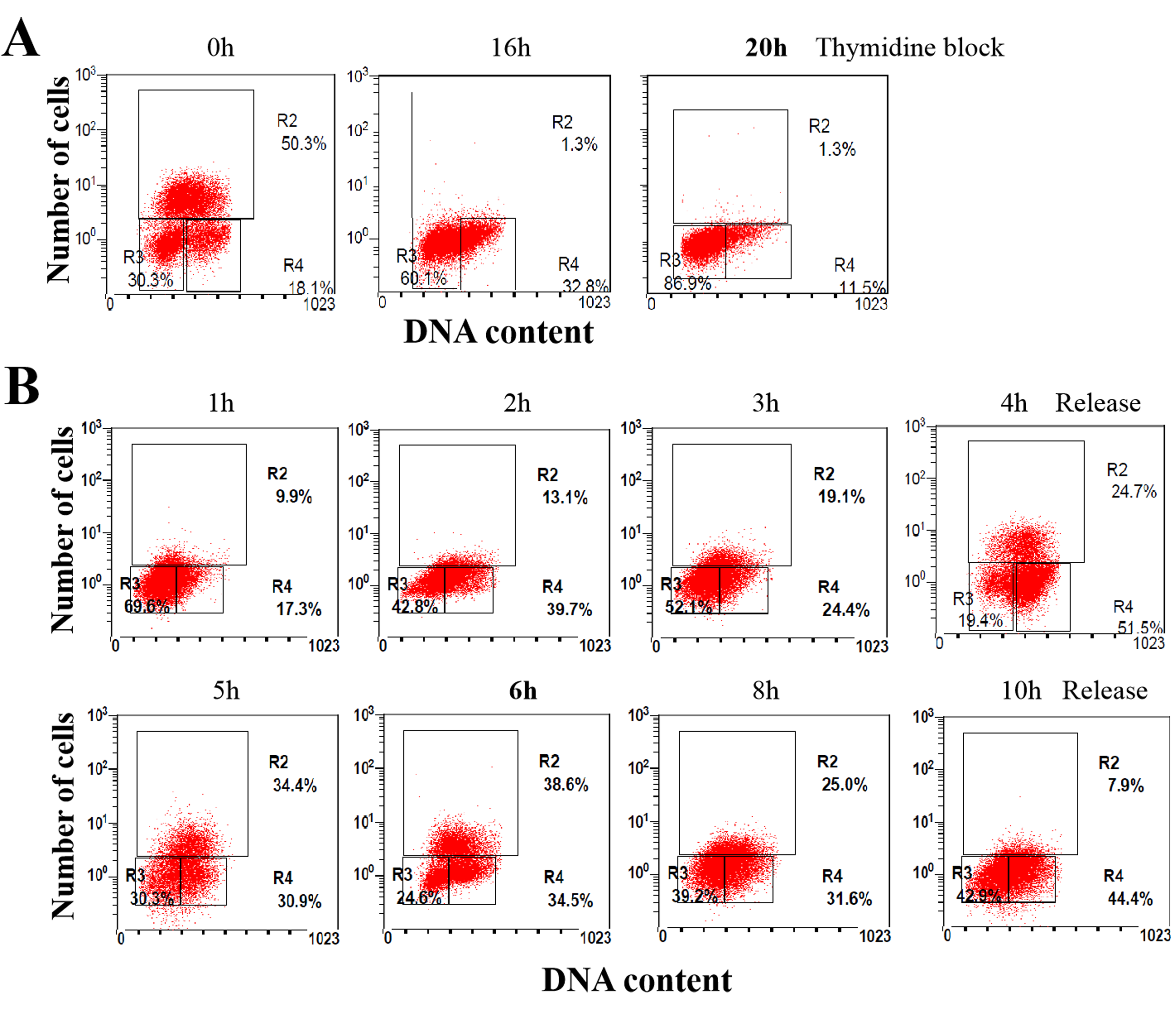

Supplement: Supplementary file 1 — Additional file 1: Figure 1. Time selection of thymidine block and release of cell cycle. a MCF-7 cells were blocked with thymidine for 0h, 20h and 24h. b After thymidine block for 20h, MCF-7 cells were released for 1h, 2h, 3h, 4h, 5h, 6h, 8h, 10h. [file 13578_2020_524_MOESM1_ESM.tif]

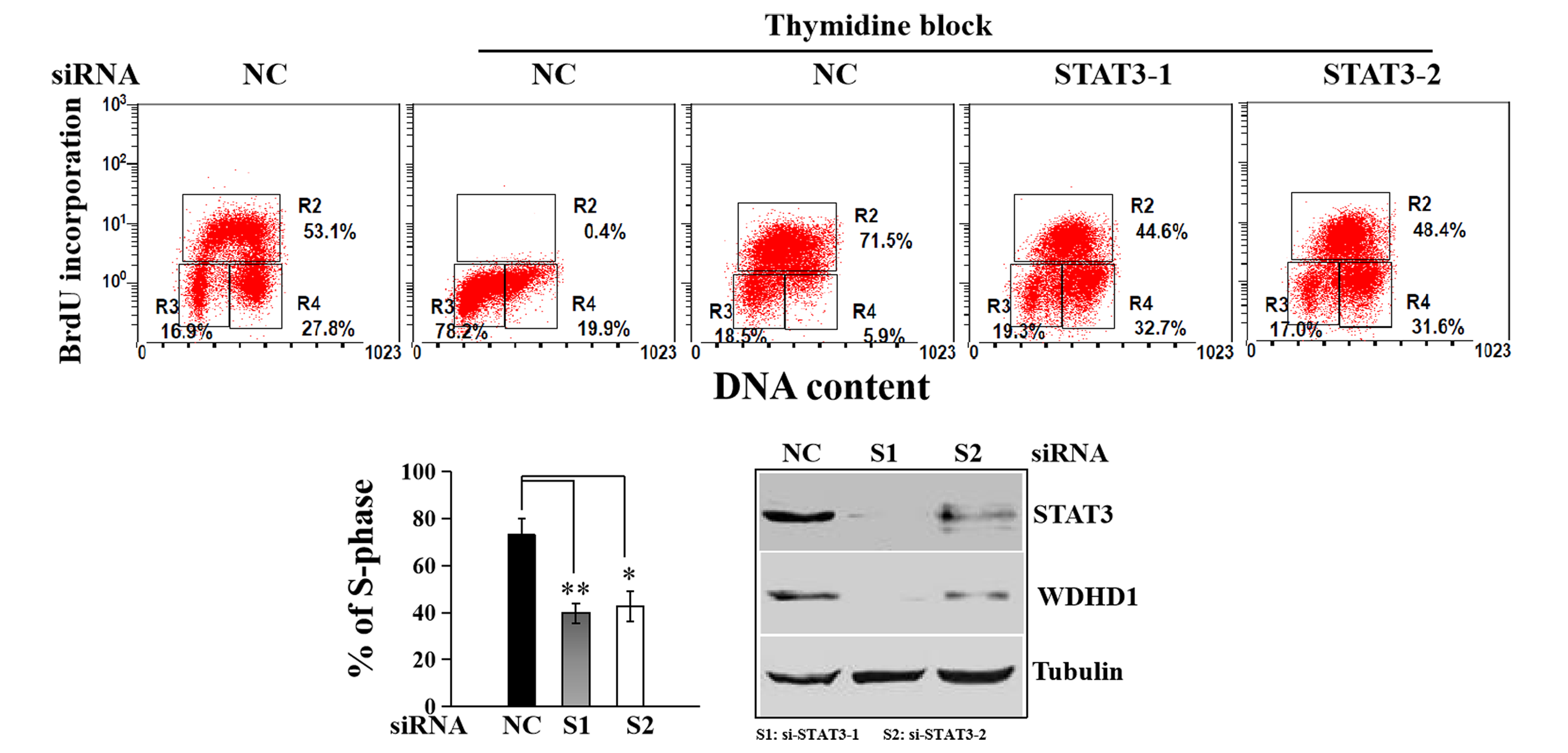

Supplement: Supplementary file 2 — Additional file 2: Figure 2. STAT3 plays a role in DNA replication in HeLa cells. After thymidine block, HeLa cells were transfected with siRNAs targeting STAT3. After releasing, cells were stained with BrdU and analyzed by flow cytometry. Data from a representative experiment of 3 were shown (Upper panel) and summarized (Lower panel). Western blots were performed using transfected cell extracts without thymidine treatment. [file 13578_2020_524_MOESM2_ESM.tif]

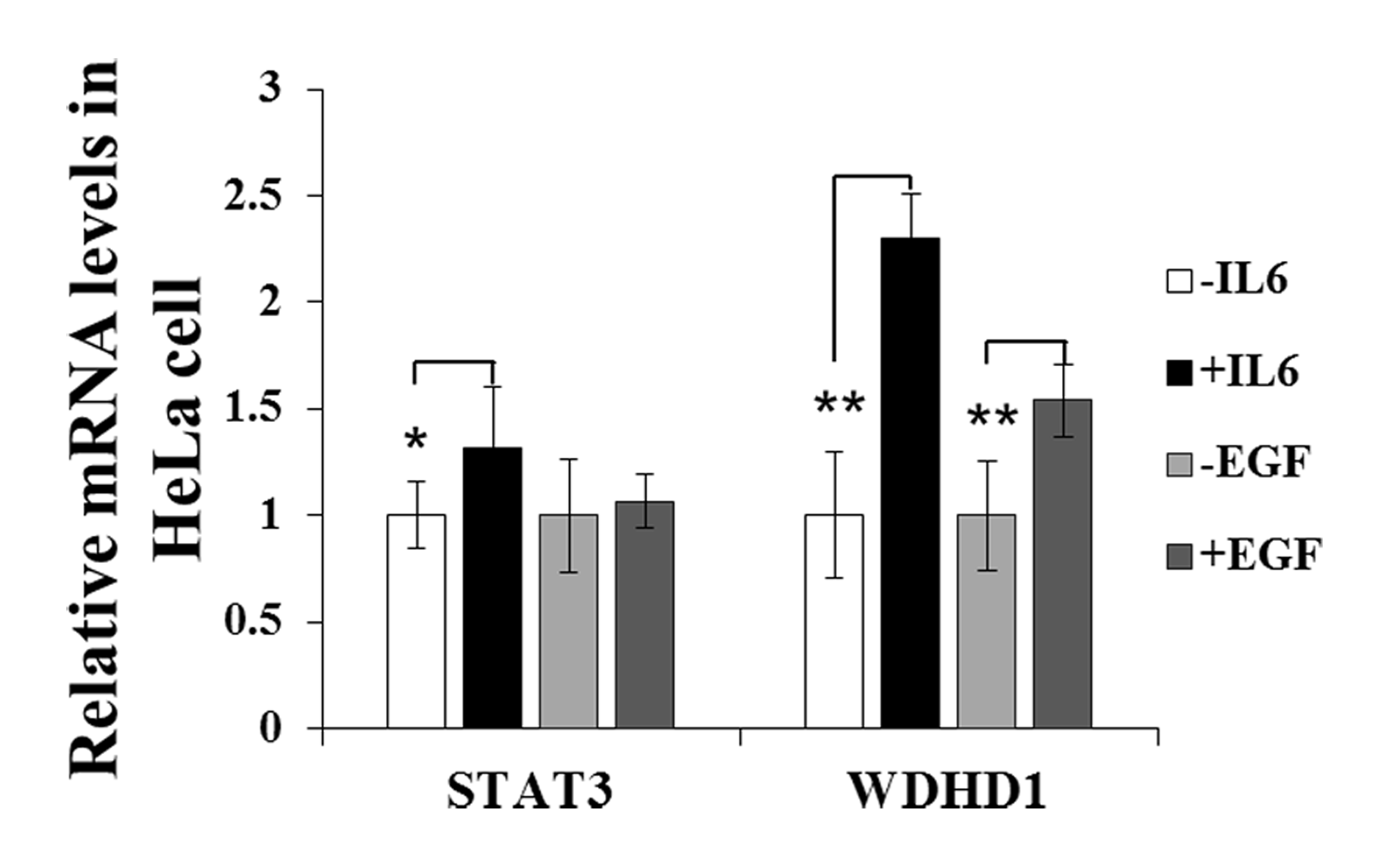

Supplement: Supplementary file 3 — Additional file 3: Figure 3. WDHD1 mRNA expression is regulated by STAT3 in HeLa cells. STAT3 and WDHD1 mRNA levels in IL-6 or EGF treated HeLa cells determined by real-time-PCR analysis. Data from a representative experiment of 3 were shown. Error bars reflect the standard deviations of the mean. *p < 0.05 **p < 0.01. NC negative control. [file 13578_2020_524_MOESM3_ESM.tif]

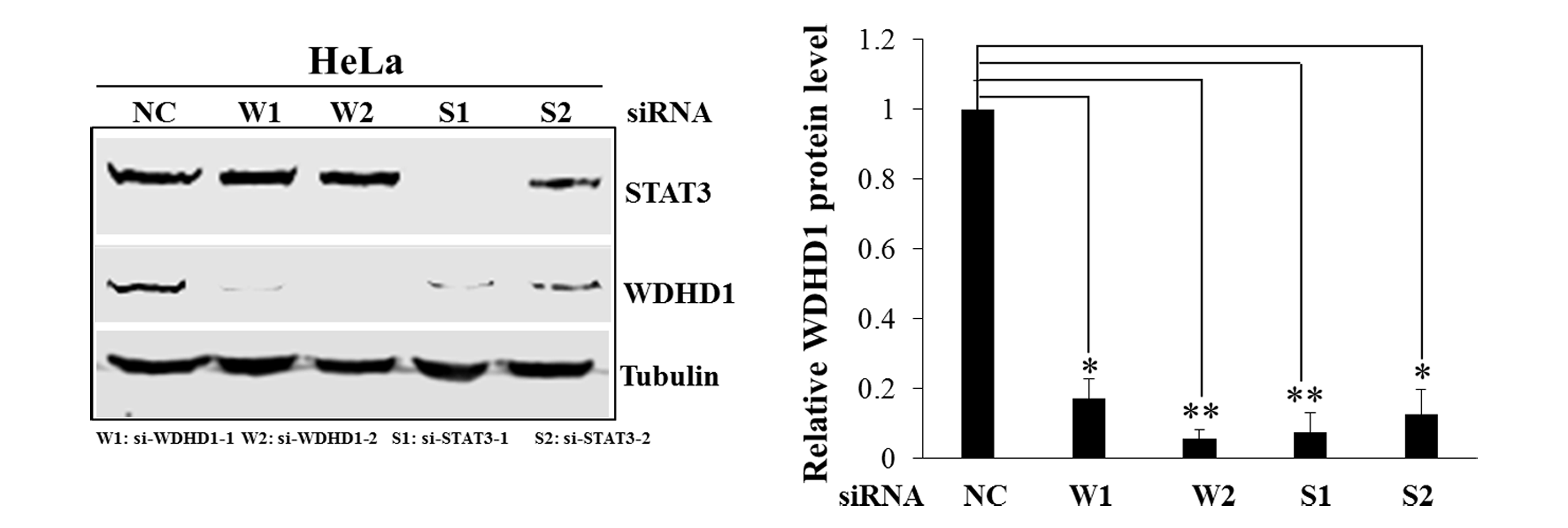

Supplement: Supplementary file 4 — Additional file 4: Figure 4. The steady-state levels of WDHD1 is regulated by STAT3 in HeLa cells. a STAT3 and WDHD1 protein levels in HeLa cells were examined by Western blotting after siRNA transfection (Left panel). Data were summarized (Right panel). Data from a representative experiment of 3 were shown. Error bars reflect the standard deviations of the mean. *p < 0.05 **p < 0.01. NC negative control. [file 13578_2020_524_MOESM4_ESM.tif]

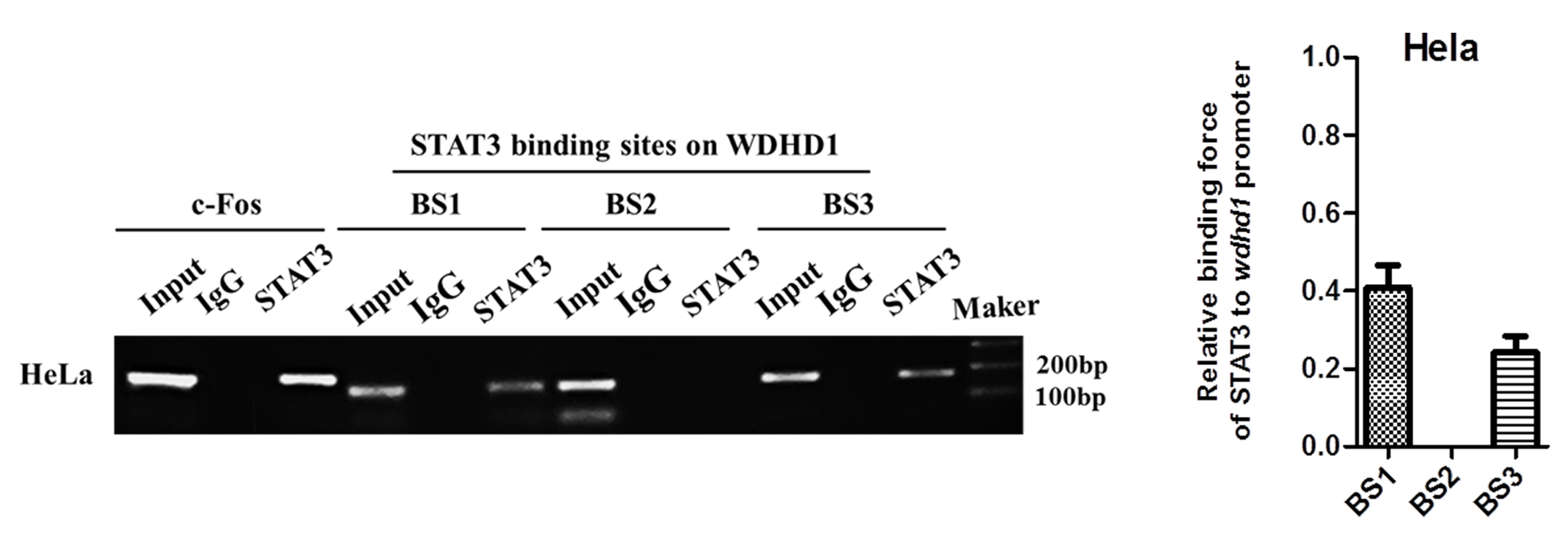

Supplement: Supplementary file 5 — Additional file 5: Figure 5. STAT3 binds to WDHD1 promoter/up regulatory region in HeLa cells. Immunoprecipitations were performed using anti-STAT3 or control IgG antibodies. PCR was performed with c-Fos or WDHD1 primers. Data from a representative experiment of 3 were shown (Left panel) and summarized (Right panel). [file 13578_2020_524_MOESM5_ESM.tif]
